# Supplementary material for: MetaRibo-Seq measures translation in microbiomes
Source: Nat Commun. 2020 Jun 29;11:3268. doi: 10.1038/s41467-020-17081-z (PMC7324362; doi:10.1038/s41467-020-17081-z)
Supplement: Supplementary file 10 — Supplementary Data 7 [file 41467_2020_17081_MOESM10_ESM.zip › File2/Confidence_High_Taxonomy/306507_out.krona.html]

Javascript must be enabled to view this page.

members
magnitude
magnitudeUnassigned
count
unassigned
taxon
rank

306507\_out

78

2
superkingdom
78

phylum
1239
78

186801
class
78

186802
order
78

family
186803
78

78
genus
841

species
1263104

SRS049900\_contig\_number\_19993SRS145497\_contig\_number\_5626
2

76
species
166486

SRS013158\_contig\_number\_4850SRS013940\_contig\_number\_13090SRS013951\_contig\_number\_32775SRS014287\_contig\_number\_1195SRS014412\_contig\_number\_6047SRS014459\_contig\_number\_10532SRS014613\_contig\_number\_contig-100\_114.105573SRS014855\_contig\_number\_14450SRS014923\_contig\_number\_contig-100\_1453.225281SRS014979\_contig\_number\_contig-100\_45684.90392SRS015065\_contig\_number\_28167SRS015190\_contig\_number\_15469SRS015264\_contig\_number\_16865SRS015431\_contig\_number\_78318SRS015578\_contig\_number\_30441SRS015579\_contig\_number\_24607SRS015854\_contig\_number\_7895SRS015890\_contig\_number\_5844SRS015960\_contig\_number\_contig-100\_102.136409SRS016267\_contig\_number\_contig-100\_83.37354SRS017821\_contig\_number\_12303SRS018984\_contig\_number\_19837SRS019068\_contig\_number\_23668SRS019397\_contig\_number\_12806SRS019787\_contig\_number\_contig-100\_3580.35948SRS024549\_contig\_number\_4610SRS043001\_contig\_number\_contig-100\_1936.54711SRS043411\_contig\_number\_12308SRS043701\_contig\_number\_9455SRS045739\_contig\_number\_3194SRS050422\_contig\_number\_contig-100\_752.175851SRS050925\_contig\_number\_9564SRS051031\_contig\_number\_contig-100\_22566.22566SRS052027\_contig\_number\_12292SRS053335\_contig\_number\_2086SRS054956\_contig\_number\_10710SRS055017\_contig\_number\_30040SRS056519\_contig\_number\_10564SRS057717\_contig\_number\_contig-100\_25542.25542SRS058070\_contig\_number\_30325SRS062427\_contig\_number\_6312SRS063518\_contig\_number\_13685SRS064557\_contig\_number\_contig-100\_26936.26936SRS065504\_contig\_number\_7939SRS075341\_contig\_number\_23773SRS075716\_contig\_number\_4037SRS076756\_contig\_number\_2825SRS077454\_contig\_number\_18143SRS077552\_contig\_number\_contig-100\_39349.80959SRS098061\_contig\_number\_9520SRS098827\_contig\_number\_12962SRS098881\_contig\_number\_21900SRS104084\_contig\_number\_14600SRS1041130\_contig\_number\_3229SRS104485\_contig\_number\_contig-100\_2398.38512SRS104975\_contig\_number\_4583SRS105153\_contig\_number\_28925SRS1055034\_contig\_number\_12845SRS1055095\_contig\_number\_contig-100\_17140.17141SRS142503\_contig\_number\_16388SRS142781\_contig\_number\_11310SRS143070\_contig\_number\_14701SRS143372\_contig\_number\_contig-100\_21684.21685SRS143417\_contig\_number\_9288SRS143722\_contig\_number\_contig-100\_194.61366SRS143780\_contig\_number\_23418SRS144362\_contig\_number\_30124SRS144537\_contig\_number\_39573SRS144714\_contig\_number\_21840SRS147919\_contig\_number\_4199SRS148091\_contig\_number\_contig-100\_400.46645SRS148159\_contig\_number\_49621SRS148424\_contig\_number\_20574SRS148874\_contig\_number\_contig-100\_40197.40198SRS149181\_contig\_number\_9777SRS149879\_contig\_number\_22181
